# Supplementary material for: The role of point-of-care ultrasound in the assessment of schistosomiasis-induced liver fibrosis: A systematic scoping review
Source: PLoS Negl Trop Dis. 2024 Mar 20;18(3):e0012033. doi: 10.1371/journal.pntd.0012033 (PMC10954168; doi:10.1371/journal.pntd.0012033)
Supplement: S1 Table — This contains the breakdown of hits for each search term and the full search string in each database that was searched. (DOCX) [file pntd.0012033.s002.docx]

| **PubMed/MEDLINE** | | |  |
| --- | --- | --- | --- |
| Search number | | Search term | Number of results |
| 1 | | (Schistosom* OR Bilharzia* OR snail* fever) | 41671 |
| 2 | | fibrosis | 372031 |
| 3 | | (periportal* OR portal OR liver* OR Symmer’s) | 1390392 |
| 4 | | 2 and 3 | 132453 |
| 5 | | 1 and 2 and 3 | 1925 |
| 6 | | (ultraso* OR sono*) | 629743 |
| 7 | | 1 and 2 and 3 and 6 | 325 |
|  | |  |  |
| **Embase** | |  |  |
| Search number | | Search term | Number of results |
| 1 | | (Schistosom* OR Bilharzia* OR snail* fever).mp. | 38794 |
| 2 | | fibrosis | 88814 |
| 3 | | exp fibrosis | 316667 |
| 4 | | (periportal* OR portal OR liver* OR Symmer’s).mp. | 1786947 |
| 5 | | 3 and 4 | 89014 |
| 6 | | 1 and 3 and 4 | 1458 |
| 7 | | (ultraso* OR sono*).mp. | 813345 |
| 8 | | 1 and 3 and 4 and 7 | 319 |
| *[mp=title, abstract, heading word, drug trade name, original title, device manufacturer, drug manufacturer, device trade name, keyword heading word, floating subheading word, candidate term word]* | | | |
|  |  | | |
| **Global Health** | | |  |
| Search number | Search term | | Number of results |
| 1 | (Schistosom* OR Bilharzia* OR snail* fever).mp. | | 35998 |
| 2 | fibrosis.mp. | | 30659 |
| 3 | exp fibrosis | | 16114 |
| 4 | (periportal* OR portal OR liver* OR Symmer’s).mp. | | 222348 |
| 5 | 3 and 4 | | 9596 |
| 6 | 1 and 3 and 4 | | 815 |
| 7 | (ultraso* OR sono*).mp. | | 38051 |
| 8 | 1 and 3 and 4 and 7 | | 175 |

**Number of hits from each element of the search strategy across six databases**

| **Global Index Medicus** | |  |
| --- | --- | --- |
| Search number | Search term | Number of results |
| 1 | (Schistosom* OR Bilharzia* OR snail* fever) | 45759 |
| 2 | fibrosis | 21905 |
| 3 | (periportal* OR portal OR liver* OR Symmer’s) | 87979 |
| 4 | 2 and 3 | 9502 |
| 5 | 1 and 2 and 3 | 343 |
| 6 | (ultraso* OR sono*) | 83639 |
| 7 | 1 and 2 and 3 and 6 | 59 |
|  |  |  |
| **Web of Science Core Collection** | |  |
| Search number | Search term | Number of results |
| 1 | (Schistosom* OR Bilharzia* OR snail* fever) | 36427 |
| 2 | fibrosis | 298954 |
| 3 | (periportal* OR portal OR liver* OR Symmer’s) | 1600623 |
| 4 | 2 and 3 | 70346 |
| 5 | 1 and 2 and 3 | 1295 |
| 6 | (ultraso* OR sono*) | 816631 |
| 7 | 1 and 2 and 3 and 6 | 300 |
|  |  |  |
| **The Cochrane Central Register of Controlled Trials** | |  |
| Search number | Search term | Number of results |
| 1 | (Schistosom* OR Bilharzia* OR snail* fever) | 763 |
| 2 | fibrosis | 16027 |
| 3 | (periportal* OR portal OR liver* OR Symmer’s) | 62262 |
| 4 | 2 and 3 | 4944 |
| 5 | 1 and 2 and 3 | 48 |
| 6 | (ultraso* OR sono*) | 44193 |
| 7 | 1 and 2 and 3 and 6 | 22 |
