# Supplementary material for: The role of point-of-care ultrasound in the assessment of schistosomiasis-induced liver fibrosis: A systematic scoping review
Source: PLoS Negl Trop Dis. 2024 Mar 20;18(3):e0012033. doi: 10.1371/journal.pntd.0012033 (PMC10954168; doi:10.1371/journal.pntd.0012033)
Supplement: S2 Text — References for all studies extracted as part of the review. (DOCX) [file pntd.0012033.s004.docx]

**Reference list for Supplementary Table S1**

1. Russell HJ, Penney JMS, Linder C, Joekes EC, Bustinduy AL, Stothard JR, et al. A cross-sectional study of periportal fibrosis and Schistosoma mansoni infection among school-aged children in a hard-to-reach area of Madagascar. Trans R Soc Trop Med Hyg. 2020;114: 315–322. doi:10.1093/trstmh/traa009
2. Song L, Wu X, Zhang B, Liu J, Ning A, Wu Z. A cross-sectional survey comparing a free treatment program for advanced schistosomiasis japonica to a general assistance program. J Parasitol Res. 2017;116: 2901–2909. doi:10.1007/s00436-017-5596-6
3. Wu YM, Xu N, Hu JY, Xu XF, Wu WX, Gao SX, et al. A simple noninvasive index to predict significant liver fibrosis in patients with advanced schistosomiasis japonica. Parasitol Int. 2013;62: 283–8. doi:10.1016/j.parint.2013.02.005
4. Espírito Santo MC, Azeredo LM, Teles HM, Gryschek RC, Ferreira CS, Amato Neto V. Abdominal ultrasound in the evaluation of fibrosis and portal hypertension in an area of schistosomiasis low endemicity. Rev Inst Med Trop Sao Paulo. 2008;50: 117–9. doi:10.1590/s0036-46652008000200010
5. Duarte DB, Vanderlei LA, de Azevêdo Bispo RK, Pinheiro ME, da Silva Junior GB, De Francesco Daher E. Acute kidney injury in schistosomiasis: a retrospective cohort of 60 patients in Brazil. J Parasitol. 2015;101: 244–7. doi:10.1645/13-361.1
6. Hua H, Yin A, Xu M, Zhou Z, You L, Guo H. Advanced schistosomiasis reappeared after curing seemingly being cured for over 20 years and without known history of reexposure to Schistosoma japonicum. J Parasitol. 2015;114: 3535–3538. doi:10.1007/s00436-015-4616-7
7. Domingues AL, Lima AR, Dias HS, Leao GC, Coutinho A. An ultrasonographic study of liver fibrosis in patients infected with Schistosoma mansoni in north-east Brazil. Trans R Soc Trop Med Hyg. 1993;87: 555–8. doi:10.1016/0035-9203(93)90087-7
8. Zeng Q, Hou J, He Y, Luo X, Zhang S, Shu H, et al. Analysis on morbidity and chemotherapy effects of Schistosoma japonicum infection in fishermen on Dongting Lake. Zhongguo Ji Sheng Chong Xue Yu Ji Sheng Chong Bing Za Zhi. 2004;22: 199–203.
9. Rodrigues ML, da Luz TPSR, Pereira CLD, Batista AD, Domingues ALC, Silva RO, et al. Assessment of periportal fibrosis in Schistosomiasis mansoni patients by proton nuclear magnetic resonance-based metabonomics models. World J Hepatol. 2022;14: 719–728. doi:10.4254/wjh.v14.i4.719
10. Jia T.-W., Zhou X.-N., Wang X.-H., Utzinger J., Steinmann P., Wua X.-H. Assessment of the age-specific disability weight of chronic schistosomiasis japonica. Bull World Health Org. 2007;85: 458–465. doi:10.2471/BLT.06.033035
11. Dias HS, Domingues AL, Cordeiro FT, Jucá N, Lopes EP. Associating portal congestive gastropathy and hepatic fibrosis in hepatosplenic mansoni schistosomiasis. Acta Trop. 2013;126: 240–3. doi:10.1016/j.actatropica.2013.02.011
12. Liu Y, Zhang P, Li J, Li H, Zhou C, Zhang Y, et al. Association between serum lipid profile and liver fibrosis in patients infected with Schistosoma japonicum. Parasit Vectors. 2022;15: 268. doi:10.1186/s13071-022-05359-8
13. Zhou LY, Zhang LF, Wu YM, Han SY. Association between serum vitamin D level and immune imbalance in advanced schistosomiasis patients with liver fibrosis. Zhongguo Xue Xi Chong Bing Fang Zhi Za Zhi. 2021;33: 22–27. doi:10.16250/j.32.1374.2020316
14. Franco KGS, de Amorim FJR, Santos MA, Rollemberg CVV, de Oliveira FA, França AVC, et al. Association of IL-9, IL-10, and IL-17 Cytokines With Hepatic Fibrosis in Human Schistosoma mansoni Infection. Front Immunol. 2021;12: 779534. doi:10.3389/fimmu.2021.779534
15. Homeida MA, el Tom I, Nash T, Bennett JL. Association of the therapeutic activity of praziquantel with the reversal of Symmers’ fibrosis induced by Schistosoma mansoni. Am J Trop Med Hyg. 1991;45: 360–5. doi:10.4269/ajtmh.1991.45.360
16. de Jesus AR, Magalhães A, Miranda DG, Miranda RG, Araújo MI, de Jesus AA, et al. Association of type 2 cytokines with hepatic fibrosis in human Schistosoma mansoni infection. Infect Immun. 2004;72: 3391–7. doi:10.1128/IAI.72.6.3391-3397.2004
17. Wiegand R, Secor W, Fleming F, French M, King C, Deol A, et al. Associations between infection intensity categories and morbidity prevalence in school-age children are much stronger for Schistosoma haematobium than for S. mansoni. PLoS Negl Trop Dis. 2021;15. doi:10.1371/journal.pntd.0009444
18. Scortegagna Junior E, Leão AR de S, Santos JEM, Sales DM, Shigueoka DC, Aguiar LAK de, et al. Avaliação da concordância entre ressonância magnética de ultra-sonografia na classificação de fibrose periportal em esquitossomóticos, segundo a classificação de Niamey. Radiol Bras. 2007;40: 303–308.
19. Meurs L, Mbow M, Vereecken K, Menten J, Mboup S, Polman K. Bladder morbidity and hepatic fibrosis in mixed Schistosoma haematobium and S. mansoni Infections: a population-wide study in Northern Senegal. PLoS Negl Trop Dis. 2012;6: e1829. doi:10.1371/journal.pntd.0001829
20. Ross AG, Olveda RM, Chy D, Olveda DU, Li Y, Harn DA, et al. Can mass drug administration lead to the sustainable control of schistosomiasis? J Infect Dis. 2015;211: 283–9. doi:10.1093/infdis/jiu416
21. Li Y, Zeng Q, Ellis MK, Xiong T, Balen J, McManus DP. CD4+ T-cell counts, CD4+/CD8+ T-cell count ratios, and antibody levels in migrant fishermen infected with Schistosoma japonicum in the Dongting Lake, China. Am J Trop Med Hyg. 2006;75: 910–3.
22. Sun T, Li G, Chen M, Nie H, Liao G, Gong Q. Change of the Vα24 NKT cells in peripheral blood of the patients with advanced schistosomiasis and its relation to the degree of hepatic fibrosis. Zhongguo Ji Sheng Chong Xue Yu Ji Sheng Chong Bing Za Zhi. 2014;32: 348–351.
23. Cai W, Chen Z, Chen F, Zhou C, Liu R, Wang J. Changes of ultrasonography and two serum biochemical indices for hepatic fibrosis in schistosomiasis japonica patients one year after praziquantel treatment. Chin Med J (Engl). 1997;110: 797–800.
24. Hu F, Gao Z-L, Yuan M, Li Z-J, Li Y-F, Liu Y-M, et al. Changing trends of schistosome infection and liver fibrosis among residents in the Poyang Lake region. Zhongguo Ji Sheng Chong Xue Yu Ji Sheng Chong Bing Za Zhi. 2021;39: 629–636.
25. Abdel-Wahab MF, Esmat G, Milad M, Abdel-Razek S, Strickland GT. Characteristic sonographic pattern of schistosomal hepatic fibrosis. Am J Trop Med Hyg. 1989;40: 72–6. doi:10.4269/ajtmh.1989.40.72
26. Chatterjee S, Mbaye A, Alfidja AT, Weyler J, Scott JT, Van Damme P, et al. Circulating levels of the neuropeptide hormone somatostatin may determine hepatic fibrosis in Schistosoma mansoni infections. Acta Trop. 2004;90: 191–203. doi:10.1016/j.actatropica.2003.12.002
27. Tabios I.K.B., Sato M.O., Tantengco O.A.G., Fornillos R.J.C., Kirinoki M., Sato M., et al. Circulating microRNAs as Biomarkers of Hepatic Fibrosis in Schistosomiasis Japonica Patients in the Philippines. Diagnostics (Basel). 2022;12: 1902. doi:10.3390/diagnostics12081902
28. Ravera M, Reggiori A, Cocozza E, Ciantia F, Riccioni G. Clinical and endoscopic aspects of hepatosplenic schistosomiasis in Uganda. Eur J Gastroenterol Hepatol. 1996;8: 693–7.
29. Ruiz-Guevara R, de Noya BA, Valero SK, Lecuna P, Garassini M, Noya O. Clinical and ultrasound findings before and after praziquantel treatment among Venezuelan schistosomiasis patients. Rev Soc Bras Med Trop. 2007;40: 505–11. doi:10.1590/s0037-86822007000500003
30. Atef M. ES, Mohammad S, Alaa G, Ahmad N. N, Ayman T.A. M, Magdy A.S. A, et al. Clinico-epidemiological study of human fascioliasis in an endemic focus in Dakahlia governorate, Egypt. J Egypt Soc Parasitol. 2001;31: 725–736.
31. Abebe N, Erko B, Medhin G, Berhe N. Clinico-epidemiological study of Schistosomiasis mansoni in Waja-Timuga, District of Alamata, northern Ethiopia. Parasit Vectors. 2014;7: 158. doi:10.1186/1756-3305-7-158
32. Magalhães TV, Gazzinelli G, Alvarez MC, Lima e Silva FC, Fraga LA, Silveira AM, et al. Comparative clinical and ultrasound study of egg-negative and egg-positive individuals from Schistosoma mansoni low morbidity endemic areas, and hospitalized patients with hepatosplenic disease. Rev Soc Bras Med Trop. 2005;38: 33–7. doi:10.1590/s0037-86822005000100007
33. Prata A, Ruiz-Guevara R, Antunes CM, Marinho CC, Queiroz LC, Voieta I, et al. Comparison between clinical and ultrasonographic findings in cases of periportal fibrosis in an endemic area for schistosomiasis mansoni in Brazil. Rev Soc Bras Med Trop. 2010;43: 129–34. doi:10.1590/s0037-86822010000200005
34. Andrianah GEP, Rakotomena D, Rakotondrainibe A, Rajaonarison Ny Ony LHN, Ranoharison HD, Ratsimba HR, et al. Contribution of Ultrasonography in the Diagnosis of Periportal Fibrosis Caused by Schistosomiasis. J Med Ultrasound. 2020;28: 41–43. doi:10.4103/JMU.JMU_16_19
35. Wu Y-M, Xu X-F, Wu W-L, Wu W-X, Gao S-X, Zhu W-J. Correlation between levels of liver fibrosis and liver fibrosis biochemical parameters of advanced schistosomiasis patients. Zhongguo Xue Xi Chong Bing Fang Zhi Za Zhi. 2014;26: 65–66, 77.
36. Medeiros TB, Domingues AL, Luna CF, Lopes EP. Correlation between platelet count and both liver fibrosis and spleen diameter in patients with schistosomiasis mansoni. Arq Gastroenterol. 2014;51: 34–8. doi:10.1590/s0004-28032014000100008
37. M.A. S. Correlation between the degree of periportal thickening, portal blood flow and splenic volume. Sci Med J. 1997;9: 13–21.
38. Abdel-Ghaffar Y, Raouf AA, Hasanien B, Hussein MH, Naiem A, El-Sherbini A. Could we diagnose active schistosomiasis in children by abdominal sonography? J Trop Pediatr. 1996;42: 374–375. doi:10.1093/tropej/42.6.374
39. Ge H, Bai D, Xia X, Xu S, Luo F, Ren G. Curative effect of Ruangan pills in treatment of schistosomiasis liver fibrosis. Zhongguo Xue Xi Chong Bing Fang Zhi Za Zhi. 2014;26: 197–199, 202.
40. Alves Oliveira LF, Moreno EC, Gazzinelli G, Martins-Filho OA, Silveira AM, Gazzinelli A, et al. Cytokine production associated with periportal fibrosis during chronic schistosomiasis mansoni in humans. Infect Immun. 2006;74: 1215–21. doi:10.1128/IAI.74.2.1215-1221.2006
41. Magalhães A, Miranda DG, Miranda RG, Araújo MI, Jesus AA, Silva A, et al. Cytokine profile associated with human chronic schistosomiasis mansoni. Mem Inst Oswaldo Cruz. 2004;99: 21–6. doi:10.1590/s0074-02762004000900004
42. Henri S, Chevillard C, Mergani A, Paris P, Gaudart J, Camilla C, et al. Cytokine regulation of periportal fibrosis in humans infected with Schistosoma mansoni: IFN-gamma is associated with protection against fibrosis and TNF-alpha with aggravation of disease. J Immunol. 2002;169: 929–36. doi:10.4049/jimmunol.169.2.929
43. Talaat RM, El-Bassiouny AI, Osman AM, Yossif M, Charmy R, Al-Sherbiny MM. Cytokine secretion profile associated with periportal fibrosis in S. mansoni-infected Egyptian patients. Parasitol Res. 2007;101: 289–99. doi:10.1007/s00436-007-0478-y
44. Vennervald BJ, Kenty L, Butterworth AE, Kariuki CH, Kadzo H, Ireri E, et al. Detailed clinical and ultrasound examination of children and adolescents in a Schistosoma mansoni endemic area in Kenya: hepatosplenic disease in the absence of portal fibrosis. Trop Med Int Health. 2004;9: 461–70. doi:10.1111/j.1365-3156.2004.01215.x
45. Zheng M, Cai WM, Zhao JK, Zhu SM, Liu RH. Determination of serum levels of YKL-40 and hyaluronic acid in patients with hepatic fibrosis due to schistosomiasis japonica and appraisal of their clinical value. Acta Trop. 2005;96: 148–52. doi:10.1016/j.actatropica.2005.07.009
46. Yuan ZK, Guo ZQ, Yang YG. Determination of the thickness of the wall of portal vein trunk in patients of schistosomiasis japonica with hepatic fibrosis and its clinical significance. Zhonghua Nei Ke Za Zhi. 1994;33: 813–816.
47. Gouveia L, Santos J, Silva R, Batista A, Domingues A, Lopes E, et al. Diagnosis of coinfection by schistosomiasis and viral hepatitis B or C using H-1 NMR-based metabonomics. PLoS One. 2017;12. doi:10.1371/journal.pone.0182196
48. Homeida M, Abdel-Gadir AF, Cheever AW, Bennett JL, Arbab BM, Ibrahium SZ, et al. Diagnosis of pathologically confirmed Symmers’ periportal fibrosis by ultrasonography: a prospective blinded study. Am J Trop Med Hyg. 1988;38: 86–91. doi:10.4269/ajtmh.1988.38.86
49. Kardorff R, Mugashe C, Gabone RM, Mahlert C, Doehring E. Diagnostic value of connective tissue metabolites in Schistosoma mansoni related liver disease. Acta Trop. 1999;73: 153–64. doi:10.1016/s0001-706x(99)00022-4
50. De Arruda S.M.B., Barreto V.S.T., Do Amaral F.J. Duplex sonography study in schistosomiasis portal hypertension: Characterization of patients with and without a history of variceal bleeding. Arq Gastroenterol. 2008;45: 11–16. doi:10.1590/S0004-28032008000100003
51. Homeida MA, Fenwick A, DeFalla AA, Suliman S, Kardaman MW, el Tom I, et al. Effect of antischistosomal chemotherapy on prevalence of Symmers’ periportal fibrosis in Sudanese villages. Lancet. 1988;2: 437–40. doi:10.1016/s0140-6736(88)90424-2
52. Bang-He XU, Yan-Min X, Qin W, Xiao-Gong C a. O, Xiao-Song ZHU. Effect of Huangqi and Danshen combined with routine liver protection drugs on schistosomiasis liver fibrosis. Chinese Journal of Schistosomiasis Control. 2010;22: 613.
53. Homeida MM, el Tom IA, Sulaiman SM, Daffalla AA, Bennett JL. Efficacy and tolerance of praziquantel in patients with Schistosoma mansoni infection and Symmers’ fibrosis: a field study in the Sudan. Am J Trop Med Hyg. 1988;38: 496–8. doi:10.4269/ajtmh.1988.38.496
54. Li YS, He YK, Zeng QR, McManus DP. Epidemiological and morbidity assessment of Schistosoma japonicum infection in a migrant fisherman community, the Dongting Lake region, China. Trans R Soc Trop Med Hyg. 2003;97: 177–81. doi:10.1016/s0035-9203(03)90112-x
55. Jie Z, Cui-Yun H, Yong-Kang HE, Yi-Qi DU, Xin-Ling YU, Yuan-Yuan W, et al. Epidemiological evaluation of schistosomiasis in migrant fishermen in Dongting Lake region. Chinese Journal of Schistosomiasis Control. 2010;22: 464.
56. Ross AG, Sleigh AC, Li YS, Williams GM, Li Y, Waine GJ, et al. Epidemiological identification of Chinese individuals putatively susceptible or insusceptible to Schistosoma japonicum: a prelude to immunogenetic study of human resistance to Asian schistosomiasis. Ann Trop Med Parasitol. 1998;92: 765–74. doi:10.1080/00034989859005
57. Saad A, Homeida M, Eltom I, Nash T, Bennett J, Hassan M. Qesophageal-varices in a region of the Sudan endemic for Schistosoma-mansoni. Br J Surg. 1991;78: 1252–1253. doi:10.1002/bjs.1800781033
58. Lima LMSTB, Lacet CMC, Parise ER. Evaluation of hepatic fibrosis by elastography in patients with schistosomiasis mansoni. Trans R Soc Trop Med Hyg. 2020;114: 531–537. doi:10.1093/trstmh/traa035
59. Mwinzi PNM, Karanja DMS, Kareko I, Magak PW, Orago ASS, Colley DG, et al. Evaluation of hepatic fibrosis in persons co-infected with Schistosoma mansoni and human immunodeficiency virus 1. Am J Trop Med Hyg. 2004;71: 783–786.
60. Odongo-Aginya EI, Lakwo TL, Doehring ME. Evaluation of schistosoma mansoni morbidity one year after praziquantel treatment in rhino cAMP and obongi in west nile, Uganda. Afr J Infect Dis. 2010;4: 43–50. doi:10.4314/ajid.v4i2.55147
61. Pereira C, Santos J, Arruda R, Rodrigues M, Siqueira E, Lemos R, et al. Evaluation of schistosomiasis mansoni morbidity by hepatic and splenic elastography. Ultrasound Med Biol. 2021;47: 1235–1243. doi:10.1016/j.ultrasmedbio.2021.01.022
62. Wu S, Tseng Y, Xu N, Yin X, Xie X, Zhang L, et al. Evaluation of transient elastography in assessing liver fibrosis in patients with advanced schistosomiasis japonica. Parasitol Int. 2018;67: 302–308. doi:10.1016/j.parint.2018.01.004
63. Thomas A, Dittrich M, Kardorff R, Talla I, Mbaye A, Sow S, et al. Evaluation of ultrasonographic staging systems for the assessment of Schistosoma mansoni induced hepatic involvement. Acta Trop. 1997;68: 347–356. doi:10.1016/S0001-706X(97)00112-5
64. Frenzel K, Grigull L, Odongo-Aginya E, Ndugwa CM, Loroni-Lakwo T, Schweigmann U, et al. Evidence for a long-term effect of a single dose of praziquantel on Schistosoma mansoni-induced hepatosplenic lesions in northern Uganda. Am J Trop Med Hyg. 1999;60: 927–31. doi:10.4269/ajtmh.1999.60.927
65. Rahoud SA, Mergani A, Khamis AH, Saeed OK, Mohamed-Ali Q, Dessein AJ, et al. Factors controlling the effect of praziquantel on liver fibrosis in Schistosoma mansoni-infected patients. FEMS Immunol Med Microbiol. 2010;58: 106–12. doi:10.1111/j.1574-695X.2009.00640.x
66. Coutinho EM, Abath FG, Barbosa CS, Domingues AL, Melo MC, Montenegro SM, et al. Factors involved in Schistosoma mansoni infection in rural areas of northeast Brazil. Mem Inst Oswaldo Cruz. 1997;92: 707–15. doi:10.1590/s0074-02761997000500027
67. Li YS, Sleigh AC, Li Y, Tanner M, Dessein A, Williams GM, et al. Five-year impact of repeated praziquantel treatment on subclinical morbidity due to Schistosoma japonicum in China. Trans R Soc Trop Med Hyg. 2002;96: 438–43. doi:10.1016/s0035-9203(02)90386-x
68. Medhat A, Abdel-Aty MA, Nafeh M, Hammam H, Abdel-Samia A, Strickland GT. Foci of Schistosoma mansoni in Assiut province in middle Egypt. Trans R Soc Trop Med Hyg. 1993;87: 404–5. doi:10.1016/0035-9203(93)90013-g
69. Silva-Teixeira DN, Contigli C, Lambertucci JR, Serufo JC, Rodrigues V Jr. Gender-related cytokine patterns in sera of schistosomiasis patients with Symmers’ fibrosis. Clin Diagn Lab Immunol. 2004;11: 627–30. doi:10.1128/CDLI.11.3.627-630.2004
70. Hirayama K, Chen H, Kikuchi M, Yin T, Itoh M, Gu X, et al. Glycine-valine dimorphism at the 86th amino acid of HLA-DRB1 influenced the prognosis of postschistosomal hepatic fibrosis. J Infect Dis. 1998;177: 1682–6. doi:10.1086/515299
71. Abdel-Wahab MF, Esmat G, Farrag A, el-Boraey YA, Strickland GT. Grading of hepatic schistosomiasis by the use of ultrasonography. Am J Trop Med Hyg. 1992;46: 403–8. doi:10.4269/ajtmh.1992.46.403
72. Song-tao Y, Wei-heng H, Yi-xin P, Jin.-zhi G. Grading of schistosomiasis patients’ hepatic lesions by ultrasonography and analysis of portal vein and splenic vein haemodynamics. Chinese Journal of Schistosomiasis Control. 2013;19: 293.
73. Abou-Basha LM, Salem A, Osman M, el-Hefni S, Zaki A. Hepatic fibrosis due to fascioliasis and/or schistosomiasis in Abis 1 village, Egypt. East Mediterr Health J. 2000;6: 870–8.
74. Lin X, Chen G, Li J. Hepatic ultrasonographic imaging and serum amino acid levels in cured schistosomiasis cases. Zhongguo Ji Sheng Chong Xue Yu Ji Sheng Chong Bing Za Zhi. 1994;12: 143–146.
75. Ghaffar YA, Kamel M, Abdel Wahab MF, Dorgham LS, Saleh MS, el Deeb AS. Hepatitis B vaccination in children infected with Schistosoma mansoni: correlation with ultrasonographic data. Am J Trop Med Hyg. 1990;43: 516–9. doi:10.4269/ajtmh.1990.43.516
76. Darwish MA, Faris R, Darwish N, Shouman A, Gadallah M, El-Sharkawy MS, et al. Hepatitis c and cirrhotic liver disease in the Nile delta of Egypt: a community-based study. Am J Trop Med Hyg. 2001;64: 147–53. doi:10.4269/ajtmh.2001.64.147
77. Rouquet P, Verlé P, Kongs A, Talla I, Niang M. Hepatosplenic alterations determined by ultrasonography in a population recently infected with Schistosoma mansoni in Richard-Toll, Senegal. Trans R Soc Trop Med Hyg. 1993;87: 190–3. doi:10.1016/0035-9203(93)90487-b
78. El Scheich T, Hofer L, Kaatano G, Foya J, Odhiambo D, Igogote J, et al. Hepatosplenic morbidity due to Schistosoma mansoni in schoolchildren on Ukerewe Island, Tanzania. Parasitol Res. 2012;110: 2515–20. doi:10.1007/s00436-011-2793-6
79. Kardorff R, Olveda RM, Acosta LP, Duebbelde UJ, Aligui GD, Alcorn NJ, et al. Hepatosplenic morbidity in schistosomiasis japonica: evaluation with Doppler sonography. Am J Trop Med Hyg. 1999;60: 954–9. doi:10.4269/ajtmh.1999.60.954
80. Booth M, Vennervald B, Kabatereine N, Kazibwe F, Ouma J, Kariuki C, et al. Hepatosplenic morbidity in two neighbouring communities in Uganda with high levels of Schistosoma mansoni infection but very different durations of residence. Trans R Soc Trop Med Hyg. 2004;98: 125–136. doi:10.1016/S0035-9203(03)00018-X
81. Lambertucci JR, Cota GF, Pinto-Silva RA, Serufo JC, Gerspacher-Lara R, Costa Drummond S, et al. Hepatosplenic schistosomiasis in field-based studies: a combined clinical and sonographic definition. Mem Inst Oswaldo Cruz. 2001;96 Suppl: 147–50. doi:10.1590/s0074-02762001000900022
82. Richter J, Zwingenberger K, Ali QM, Lima Wde M, Dacal AR, de Siqueira GV, et al. Hepatosplenic schistosomiasis: comparison of sonographic findings in Brazilian and Sudanese patients--correlation of sonographic findings with clinical symptoms. Radiology. 1992;184: 711–6. doi:10.1148/radiology.184.3.1509054
83. Silveira AM, Bethony J, Gazzinelli A, Kloos H, Fraga LA, Alvares MC, et al. High levels of IgG4 to Schistosoma mansoni egg antigens in individuals with periportal fibrosis. Am J Trop Med Hyg. 2002;66: 542–9. doi:10.4269/ajtmh.2002.66.542
84. Mutengo MM, Mwansa JC, Mduluza T, Sianongo S, Chipeta J. High Schistosoma mansoni disease burden in a rural district of western Zambia. Am J Trop Med Hyg. 2014;91: 965–72. doi:10.4269/ajtmh.13-0612
85. Hirayama K, Chen H, Kikuchi M, Yin T, Gu X, Liu J, et al. HLA-DR-DQ alleles and HLA-DP alleles are independently associated with susceptibility to different stages of post-schistosomal hepatic fibrosis in the Chinese population: HLA-class II associated with schistosomal liver fibrosis. Tissue Antigens. 1999;53: 269–274. doi:10.1034/j.1399-0039.1999.530307.x
86. Pascal M, Abdallahi OM, Elwali NE, Mergani A, Qurashi MA, Magzoub M, et al. Hyaluronate levels and markers of oxidative stress in the serum of Sudanese subjects at risk of infection with Schistosoma mansoni. Trans R Soc Trop Med Hyg. 2000;94: 66–70. doi:10.1016/s0035-9203(00)90443-7
87. Chevillard C, Moukoko CE, Elwali NE, Bream JH, Kouriba B, Argiro L, et al. IFN-gamma polymorphisms (IFN-gamma +2109 and IFN-gamma +3810) are associated with severe hepatic fibrosis in human hepatic schistosomiasis (Schistosoma mansoni). J Immunol. 2003;171: 5596–601. doi:10.4049/jimmunol.171.10.5596
88. Nassr AKh, Hassan MM, Abdel Salam FM, Lashin AH, Shahin WA, Amin H. IgG isotypes in schistosomiasis patients before and after praziquantel. J Egypt Soc Parasitol. 2002;32: 931–52.
89. Voieta I, de Queiroz LC, Andrade LM, Silva LC, Fontes VF, Barbosa A Jr, et al. Imaging techniques and histology in the evaluation of liver fibrosis in hepatosplenic schistosomiasis mansoni in Brazil: a comparative study. Mem Inst Oswaldo Cruz. 2010;105: 414–21. doi:10.1590/s0074-02762010000400011
90. Tawfeek GM, Alafifi AM, Azmy MF. Immunological indicators of morbidity in human schistosomiasis mansoni: role of vascular endothelial growth factor and anti-soluble egg antigen IgG4 in disease progression. J Egypt Soc Parasitol. 2003;33: 597–614.
91. Boghdadi G, Khalik DA, Wahab SA, Farghaly A. Immunomodulatory effect of R848 on cytokine production associated with Schistosoma mansoni infection. Parasitol Res. 2013;112: 135–40. doi:10.1007/s00436-012-3116-2
92. Ramzy I, Elsharkawy A, Fouad R, Hafez HA, El Raziky M, El Akel W, et al. Impact of old Schistosomiasis infection on the use of transient elastography (Fibroscan) for staging of fibrosis in chronic HCV patients. Acta Trop. 2017;176: 283–287. doi:10.1016/j.actatropica.2017.08.019
93. Cardoso LS, Barreto Ade S, Fernandes JS, Oliveira RR, de Souza Rda P, Carvalho EM, et al. Impaired lymphocyte profile in schistosomiasis patients with periportal fibrosis. Clin Dev Immunol. 2013;2013: 710647. doi:10.1155/2013/710647
94. de Carvalho BT, Coutinho Domingues AL, de Almeida Lopes EP, Brandão SC. Increased Hepatic Arterial Blood Flow Measured by Hepatic Perfusion Index in Hepatosplenic Schistosomiasis: New Concepts for an Old Disease. Dig Dis Sci. 2016;61: 2118–26. doi:10.1007/s10620-016-4080-y
95. Yu X-L, Zhou J, He Y-K, Huang M-Z, Li Y-S. Influence factors of Schistosoma japonicum infection among fishermen in eastern Dongting Lake Region. Zhongguo Ji Sheng Chong Xue Yu Ji Sheng Chong Bing Za Zhi. 2013;31: 307–309, 314.
96. Silva P.C.V., Gomes A.V., De Britto L.R.P.B., De Lima E.L.S., Da Silva J.L., Montenegro S.M.L., et al. Influence of a TNF-alpha Polymorphism on the Severity of Schistosomiasis Periportal Fibrosis in the Northeast of Brazil. Gen Test Mol Biomarkers. 2017;21: 658–662. doi:10.1089/gtmb.2017.0133
97. Doehring-Schwerdtfeger E, Kaiser C, Franke D, Kardorff R, Ali Q, Abdelrahim I. Interobserver variance in ultrasonographical assessment of schistosoma-mansoni-related morbidity in young schoolchildren. Acta Trop. 1992;51: 85–88. doi:10.1016/0001-706X(92)90022-P
98. Mueller A, Fuss A, Ziegler U, Kaatano GM, Mazigo HD. Intestinal schistosomiasis of Ijinga Island, north-western Tanzania: prevalence, intensity of infection, hepatosplenic morbidities and their associated factors. BMC Infect Dis. 2019;19: 832. doi:10.1186/s12879-019-4451-z
99. de Queiros A, Brandao S, Domingues A, Macedo L, Ourem M, Lopes E. Intrapulmonary Vascular Dilatation Evaluated by Tc-99m-MAA Scintigraphy and Its Association with Portal Hypertension in Schistosomiasis. PLoS Negl Trop Dis. 2014;8. doi:10.1371/journal.pntd.0002881
100. Chen W-Z, Xu H-L, Liu Z-C, Zhu J-H. Investigation and treatment of newly discovered advanced schistosomiasis cases in Hunan Province in 2011. Zhongguo Ji Sheng Chong Xue Yu Ji Sheng Chong Bing Za Zhi. 2013;31: 342–345.
101. Asztely MS, Eriksson B, Gabone RM, Nilsson L-Å. Is ultrasonography useful for population studies on schistosomiasis mansoni? An evaluation based on a survey on a population from Kome Island, Tanzania. Acta Radiol Open. 2016;5: 2058460116686392. doi:10.1177/2058460116686392
102. Kardorff R, Traore M, Diarra A, Sacko M, Maiga M, Franke D, et al. Lack of ultrasonographic evidence for severe hepatosplenic morbidity in schistosomiasis mansoni in Mali. Am J Trop Med Hyg. 1994;51: 190–7. doi:10.4269/ajtmh.1994.51.190
103. Isam SM, Ismail AA, Mohamed I, Suliman FS. Laparoscopic cholecystectomy in patients with bilharzial portal hypertension. JSLS. 2000;4: 155–7.
104. Berhe N, Geitung JT, Medhin G, Gundersen SG. Large scale evaluation of WHO’s ultrasonographic staging system of schistosomal periportal fibrosis in Ethiopia. Trop Med Int Health. 2006;11: 1286–94. doi:10.1111/j.1365-3156.2006.01665.x
105. Keang H, Odermatt P, Odermatt-Biays S, Cheam S, Degrémont A, Hatz C. Liver morbidity due to Schistosoma mekongi in Cambodia after seven rounds of mass drug administration. Trans R Soc Trop Med Hyg. 2007;101: 759–65. doi:10.1016/j.trstmh.2007.04.007
106. Eltoum IA, Saad AM, Ismail BM, Ali MM, Suliaman S, Bennett JL, et al. Liver sonography in an area endemic for schistosomiasis haematobium. Am J Trop Med Hyg. 1993;48: 77–81. doi:10.4269/ajtmh.1993.48.77
107. Silva CF, Nardelli MJ, Barbosa FA, Galizzi HO, Cal TCMF, Ferrari TCA, et al. Liver stiffness is able to differentiate hepatosplenic Schistosomiasis mansoni from liver cirrhosis and spleen stiffness may be a predictor of variceal bleeding in hepatosplenic schistosomiasis. Trans R Soc Trop Med Hyg. 2022;116: 26–33. doi:10.1093/trstmh/trab041
108. Carvalho Santos J, Dória Batista A, Maria Mola Vasconcelos C, Souza Lemos R, Romão de Souza Junior V, Dessein A, et al. Liver ultrasound elastography for the evaluation of periportal fibrosis in schistosomiasis mansoni: A cross-sectional study. PLoS Negl Trop Dis. 2018;12: e0006868. doi:10.1371/journal.pntd.0006868
109. Mott KE, Chen MG, Abdel-Wahab F, Burki A, Dixon H, Xu FN, et al. Liver ultrasound findings in a low prevalence area of S. japonicum in China: comparison with history, physical examination, parasitological and serological results. Acta Trop. 1992;51: 65–84. doi:10.1016/0001-706x(92)90021-o
110. Xu Q, Huang H. Long-term efficacy of sodium tanshinone ⅡA sulfonate in treatment of hepatic fibrosis induced by schistosomiasis japonica. China Tropical Medicine. 2016;16: 1216–1219. doi:10.13604/j.cnki.46-1064/r.2016.12.17
111. Mutengo MM, Mduluza T, Kelly P, Mwansa JCL, Kwenda G, Musonda P, et al. Low IL-6, IL-10, and TNF-a and high IL-13 cytokine levels are associated with severe hepatic fibrosis in Schistosoma mansoni chronically exposed individuals. J Parasitol Res. 2018;2018: Article-9754060. doi:10.1155/2018/9754060
112. Aziz IA, Yacoub M, Rashid L, Solieman A. Malondialdehyde; Lipid peroxidation plasma biomarker correlated with hepatic fibrosis in human Schistosoma mansoni infection. Acta Parasitol. 2015;60: 735–42. doi:10.1515/ap-2015-0105
113. King CH, Magak P, Salam EA, Ouma JH, Kariuki HC, Blanton RE. Measuring morbidity in schistosomiasis mansoni: relationship between image pattern, portal vein diameter and portal branch thickness in large-scale surveys using new WHO coding guidelines for ultrasound in schistosomiasis. Trop Med Int Health. 2003;8: 109–17. doi:10.1046/j.1365-3156.2003.00994.x
114. Homeida M, Ahmed S, Dafalla A, Suliman S, Eltom I, Nash T, et al. Morbidity associated with Schistosoma mansoni infection as determined by ultrasound: a study in Gezira, Sudan. Am J Trop Med Hyg. 1988;39: 196–201. doi:10.4269/ajtmh.1988.39.196
115. De Jesus AR, Miranda DG, Miranda RG, Araújo I, Magalhães A, Bacellar M, et al. Morbidity associated with Schistosoma mansoni infection determined by ultrasound in an endemic area of Brazil, Caatinga do Moura. Am J Trop Med Hyg. 2000;63: 1–4. doi:10.4269/ajtmh.2000.63.1
116. Davis SM, Wiegand RE, Mulama F, Kareko EI, Harris R, Ochola E, et al. Morbidity associated with schistosomiasis before and after treatment in young children in Rusinga Island, western Kenya. Am J Trop Med Hyg. 2015;92: 952–8. doi:10.4269/ajtmh.14-0346
117. Ndamba J, Makaza N, Kaondera KC, Munjoma M. Morbidity due to Schistosoma mansoni among sugar-cane cutters in Zimbabwe. Int J Epidemiol. 1991;20: 787–95. doi:10.1093/ije/20.3.787
118. Zaki A, Bassili A, Amin G, Aref T, Kandil M, Abou Basha LM. Morbidity of schistosomiasis mansoni in rural Alexandria, Egypt. J Egypt Soc Parasitol. 2003;33: 695–710.
119. Hoffmann H, Esterre P, Ravaoalimalala VA, Ehrich JH, Doehring E. Morbidity of schistosomiasis mansoni in the highlands of Madagascar and comparison of current sonographical classification systems. Trans R Soc Trop Med Hyg. 2001;95: 623–9. doi:10.1016/s0035-9203(01)90099-9
120. Kamdem SD, Konhawa F, Kuemkon EM, Meyo Kamguia L, Tchanana GK, Nche F, et al. Negative Association of Interleukin-33 Plasma Levels and Schistosomiasis Infection in a Site of Polyparasitism in Rural Cameroon. Front Immunol. 2019;10: 2827. doi:10.3389/fimmu.2019.02827
121. Barreto AV, Alecrim VM, Medeiros TB, Domingues AL, Lopes EP, Martins JR, et al. New index for the diagnosis of liver fibrosis in Schistosomiasis mansoni. Arq Gastroenterol. 2017;54: 51–56. doi:10.1590/S0004-2803.2017v54n1-10
122. Nardelli MJ, Veiga ZDST, Faria LC, Pereira GHS, da Silva CF, Barbosa FA, et al. Noninvasive predictors of esophageal varices in patients with hepatosplenic schistosomiasis mansoni. Acta Trop. 2022;226: 106283. doi:10.1016/j.actatropica.2021.106283
123. Coutinho HM, McGarvey ST, Acosta LP, Manalo DL, Langdon GC, Leenstra T, et al. Nutritional status and serum cytokine profiles in children, adolescents, and young adults with Schistosoma japonicum-associated hepatic fibrosis, in Leyte, Philippines. J Infect Dis. 2005;192: 528–36. doi:10.1086/430929
124. Tanabe M, Gonçalves JF, Gonçalves FJ, Tateno S, Takeuchi T. Occurrence of a community with high morbidity associated with Schistosoma mansoni infection regardless of low infection intensity in north-east Brazil. Trans R Soc Trop Med Hyg. 1997;91: 144–9. doi:10.1016/s0035-9203(97)90201-7
125. el Shiekh Mohamed AR, al Karawi MA, Yasawy MI. Organ involvement in hepato-intestinal schistosomiasis. Hepatogastroenterology. 1994;41: 370–6.
126. Yazdanpanah Y, Thomas A, Kardorff R, Talla I, Sow S, Niang M, et al. Organometric investigations of the spleen and liver by ultrasound in Schistosoma mansoni endemic and nonendemic villages in Senegal Am J Trop Med Hyg. 1997;57: 245–249. doi:10.4269/ajtmh.1997.57.245
127. Nigo M.M., Odermatt P., Nigo D.W., Salieb-Beugelaar G.B., Battegay M., Hunziker P.R. Patients with severe schistosomiasis mansoni in Ituri Province, Democratic Republic of the Congo. Infect Dis of Poverty. 2021;10: 39. doi:10.1186/s40249-021-00815-6
128. Ayé P, Phongluxa K, Vonghachack Y, Sayasone S, Oroth R, Odermatt P. Patients with severe schistosomiasis mekongi morbidity demonstrating ongoing transmission in Southern Lao People’s Democratic Republic. Acta Trop. 2020;204: 105323. doi:10.1016/j.actatropica.2019.105323
129. Mazigo HD, Dunne DW, Morona D, Lutufyo TE, Kinung’hi SM, Kaatano G, et al. Periportal fibrosis, liver and spleen sizes among S. mansoni mono or co-infected individuals with human immunodeficiency virus-1 in fishing villages along Lake Victoria shores, North-Western, Tanzania. Parasit Vectors. 2015;8: 260. doi:10.1186/s13071-015-0876-4
130. Ahmed MH, Emara MH, Elfert AA, El-Saka AM, Abd-Elsalam S, Yousef M. Persistent Colonic Schistosomiasis among Symptomatic Rural Inhabitants in the Egyptian Nile Delta. Mediterr J Hematol Infect Dis. 2021;13: e2021033. doi:10.4084/MJHID.2021.033
131. Eltoum IA, Taha TE, Saad AM, Suliman SM, Bennett JL, Nash TE, et al. Predictors of upper gastrointestinal bleeding in patients with schistosomal periportal fibrosis. Br J Surg. 1994;81: 996–9. doi:10.1002/bjs.1800810722
132. Kariuki HC, Mbugua G, Magak P, Bailey JA, Muchiri EM, Thiongo FW, et al. Prevalence and familial aggregation of schistosomal liver morbidity in Kenya: evaluation by new ultrasound criteria. J Infect Dis. 2001;183: 960–6. doi:10.1086/319247
133. el-Hawey AM, Abdel-Rahman AH, Agina AA, Amer MM, Hashem YA, Gomaa AA, et al. Prevalence and morbidity of schistosomiasis among rural fishermen at two Egyptian villages (Gharbia Governorate). J Egypt Soc Parasitol. 1995;25: 649–57.
134. Mudawi H, Ali Y, El Tahir M. Prevalence of gastric varices and portal hypertensive gastropathy in patients with Symmers periportal fibrosis. Ann Saudi Med. 2008;28: 42–4. doi:10.5144/0256-4947.2008.42
135. Kéita AD, Sangho H, Sacko M, Diarra Z, Simaga SY, Traore I. Prevalence of schistomasiasis lesions detected by ultrasonography in children in Molodo, Mali. Gastroenterol Clin Biol. 2005;29: 652–5. doi:10.1016/s0399-8320(05)82151-7
136. Opio C, Kazibwe F, Ocama P, Rejani L, Belousova E, Ajal P. Profiling lifetime episodes of upper gastrointestinal bleeding among patients from rural Sub-Saharan Africa where schistosoma mansoni is endemic. Pan Afr Med J. 2016;24. doi:10.11604/pamj.2016.24.296.9755
137. Barbosa MM, Lamounier JA, Oliveira EC, Souza MV, Marques DS, Silva AA, et al. Pulmonary hypertension in schistosomiasis mansoni. Trans R Soc Trop Med Hyg. 1996;90: 663–5. doi:10.1016/s0035-9203(96)90424-1
138. Berhe N, Halvorsen BL, Gundersen TE, Myrvang B, Gundersen SG, Blomhoff R. Reduced serum concentrations of retinol and alpha-tocopherol and high concentrations of hydroperoxides are associated with community levels of S. mansoni infection and schistosomal periportal fibrosis in Ethiopian school children. Am J Trop Med Hyg. 2007;76: 943–9.
139. Hassan MM, Hegab MH, Soliman SZ, Gaber OA, Shalaby MM, Kamel FM. Relationship between circulating antigen level and morbidity in Schistosoma mansoni-infected children evaluated by ultrasonography. Am J Trop Med Hyg. 1999;61: 635–8. doi:10.4269/ajtmh.1999.61.635
140. Leite LA, Domingues AL, Lopes EP, Ferreira Rde C, Pimenta Ade A Filho, da Fonseca CS, et al. Relationship between splenomegaly and hematologic findings in patients with hepatosplenic schistosomiasis. Rev Bras Hematol Hemoter. 2013;35: 332–6. doi:10.5581/1516-8484.20130098
141. Kaiser C, Doehring-Schwerdtfeger E, Abdel-Rahim IM, Daubner G, Vester U, Homeida MM, et al. Renal function and morphology in Sudanese patients with advanced hepatosplenic schistosomiasis and portal hypertension. Am J Trop Med Hyg. 1989;40: 176–85. doi:10.4269/ajtmh.1989.40.176
142. Santos GT, Sales DM, Leão AR de S, Santos JEM, Aguiar LAK de, Brant PE, et al. Reproducibility of ultrasonography in the assessment of periportal fibrosis according to Niamey criteria in patients with schistosomiasis mansoni. Radiol Bras. 2007;40: 377–381. doi:10.1590/S0100-39842007000600005
143. Maurizio R, Eugenio C, Roberto PR. Results of sclerotherapy for bleeding esophageal varices in patients with schistosomal liver disease. A retrospective study. Hepatogastroenterology. 2000;47: 424–8.
144. Berhe N, Myrvang B, Gundersen SG. Reversibility of schistosomal periportal thickening/fibrosis after praziquantel therapy: a twenty-six month follow-up study in Ethiopia. Am J Trop Med Hyg. 2008;78: 228–34.
145. Osman K. S, Alain J. D, Nasruddin EW, Ahmed A. A, Mubarak M, Kamal A. ES, et al. Schistosoma mansoni associated mortality in Gezira: Determined by clinical and ultrasound examination. Sudan J Public Health. 2006;1: 27–30.
146. Mazigo HD, Nuwaha F, Dunne DW, Kaatano GM, Angelo T, Kepha S, et al. Schistosoma mansoni Infection and Its Related Morbidity among Adults Living in Selected Villages of Mara Region, North-Western Tanzania: A Cross-Sectional Exploratory Study. Korean J Parasitol. 2017;55: 533–540. doi:10.3347/kjp.2017.55.5.533
147. Burchard GD, Guissé-Sow F, Diop M, Ly A, Lanuit R, Gryseels B, et al. Schistosoma mansoni infection in a recently exposed community in Senegal: lack of correlation between liver morphology in ultrasound and connective tissue metabolites in serum. Trop Med Int Health. 1998;3: 234–41. doi:10.1046/j.1365-3156.1998.00217.x
148. Malenganisho WL, Magnussen P, Friis H, Siza J, Kaatano G, Temu M, et al. Schistosoma mansoni morbidity among adults in two villages along Lake Victoria shores in Mwanza District, Tanzania. Trans R Soc Trop Med Hyg. 2008;102: 532–41. doi:10.1016/j.trstmh.2008.03.006
149. Nalugwa A, Nuwaha F, Tukahebwa EM, Olsen A. Schistosoma mansoni-Associated Morbidity among Preschool-Aged Children along the Shores of Lake Victoria in Uganda. Trop Med Infect Dis. 2017;2. doi:10.3390/tropicalmed2040058
150. Kaatano GM, Min DY, Siza JE, Yong TS, Chai JY, Ko Y, et al. Schistosoma mansoni-Related Hepatosplenic Morbidity in Adult Population on Kome Island, Sengerema District, Tanzania. Korean J Parasitol. 2015;53: 545–51. doi:10.3347/kjp.2015.53.5.545
151. Silva LC, Andrade LM, Queiroz LC, Voieta I, Azeredo LM, Antunes CM, et al. Schistosoma mansoni: magnetic resonance analysis of liver fibrosis according to WHO patterns for ultrasound assessment of schistosomiasis-related morbidity. Mem Inst Oswaldo Cruz. 2010;105: 467–70. doi:10.1590/s0074-02762010000400019
152. Blanton RE, Salam EA, Ehsan A, King CH, Goddard KA. Schistosomal hepatic fibrosis and the interferon gamma receptor: a linkage analysis using single-nucleotide polymorphic markers. Eur J Hum Genet. 2005;13: 660–8. doi:10.1038/sj.ejhg.5201388
153. Davidson RN, Houston S, Kiire CF. Schistosomal periportal fibrosis in Zimbabwe: use of ultrasound in patients with oesophageal varices. Trans R Soc Trop Med Hyg. 1991;85: 380–2. doi:10.1016/0035-9203(91)90298-d
154. Gonçalves-Macedo L, Lopes EP, Domingues ALC, Markman B Filho, Mota VG, Luna CF. Schistosomiasis and hepatopulmonary syndrome: the role of concomitant liver cirrhosis. Mem Inst Oswaldo Cruz. 2017;112: 469–473. doi:10.1590/0074-02760160383
155. Nono JK, Kamdem SD, Netongo PM, Dabee S, Schomaker M, Oumarou A, et al. Schistosomiasis Burden and Its Association With Lower Measles Vaccine Responses in School Children From Rural Cameroon. Front Immunol. 2018;9: 2295. doi:10.3389/fimmu.2018.02295
156. Wiest PM, Wu G, Zhong S, McGarvey ST, Tan E, Yuan J, et al. Schistosomiasis japonica on Jishan Island, Jiangxi Province, People’s Republic of China: persistence of hepatic fibrosis after reduction of the prevalence of infection with age. Trans R Soc Trop Med Hyg. 1993;87: 290–4. doi:10.1016/0035-9203(93)90133-b
157. Silva CC, Domingues AL, Lopes EP, Morais CN, Santos RB, Luna CF, et al. Schistosomiasis mansoni: ultrasound-evaluated hepatic fibrosis and serum concentrations of hyaluronic acid. Ann Trop Med Parasitol. 2011;105: 233–9. doi:10.1179/136485911X12987676649629
158. Köpke-Aguiar LA, Martins JR, Passerotti CC, Toledo CF, Nader HB, Borges DR. Serum hyaluronic acid as a comprehensive marker to assess severity of liver disease in schistosomiasis. Acta Trop. 2002;84: 117–26. doi:10.1016/s0001-706x(02)00136-5
159. Dessein AJ, Hillaire D, Elwali NE, Marquet S, Mohamed-Ali Q, Mirghani A, et al. Severe hepatic fibrosis in Schistosoma mansoni infection is controlled by a major locus that is closely linked to the interferon-gamma receptor gene. Am J Hum Genet. 1999;65: 709–21. doi:10.1086/302526
160. Li Y, Chen D, Ross AG, Burke ML, Yu X, Li RS, et al. Severe hepatosplenic schistosomiasis: clinicopathologic study of 102 cases undergoing splenectomy. Hum Pathol. 2011;42: 111–9. doi:10.1016/j.humpath.2010.05.020
161. Leite LA, Pimenta Filho AA, Ferreira Rde C, da Fonseca CS, dos Santos BS, Montenegro SM, et al. Splenectomy Improves Hemostatic and Liver Functions in Hepatosplenic Schistosomiasis Mansoni. PLoS One. 2015;10: e0135370. doi:10.1371/journal.pone.0135370
162. Guangjin S, Mingdao J, Qiyang L, Hui X, Jiangming H, Xiaomei Y. Study on histopathology, ultrasonography and some special serum enzymes and collagens for 38 advanced patients of schistosomiasis japonica. Acta Trop. 2002;82: 235–46. doi:10.1016/s0001-706x(02)00015-3
163. Fu X, Luo X, Hou X, He H, Zhou J, Wang Y, et al. Study on the relationship between the degree of periportal fibrosis, hepatic parenchymatous fibrosis and diameter of portal vein in Schistosoma japonicum infection. Zhongguo Ji Sheng Chong Xue Yu Ji Sheng Chong Bing Za Zhi. 2006;24: 265, 289, 308.
164. Mones A, Sherif MM, Abdel Halim RM. Superiority of rectal snip over serology in detection of schistosomiasis eradication: A pilot study. Arab J Gastroenterol. 2021;22: 52–55. doi:10.1016/j.ajg.2020.11.001
165. Mohamed-Ali Q, Elwali NE, Abdelhameed AA, Mergani A, Rahoud S, Elagib KE, et al. Susceptibility to periportal (Symmers) fibrosis in human schistosoma mansoni infections: evidence that intensity and duration of infection, gender, and inherited factors are critical in disease progression. J Infect Dis. 1999;180: 1298–306. doi:10.1086/314999
166. Barreto AVMS, Domingues ALC, Diniz GTN, Cavalcanti AMS, Lopes EP, Montenegro SML, et al. The Coutinho index as a simple tool for screening patients with advanced forms of Schistosomiasis mansoni: a validation study. Trans R Soc Trop Med Hyg. 2022;116: 19–25. doi:10.1093/trstmh/trab040
167. Hu F, Xie SY, Yuan M, Li YF, Li ZJ, Gao ZL, et al. The Dynamics of Hepatic Fibrosis Related to Schistosomiasis and Its Risk Factors in a Cohort of China. Pathogens. 2021;10. doi:10.3390/pathogens10121532
168. Tagy AH, Saker ME, Moussa AA, Kolgah A. The effect of low-dose combined oral contraceptive pills versus injectable contraceptive (Depot Provera) on liver function tests of women with compensated bilharzial liver fibrosis. Contraception. 2001;64: 173–6. doi:10.1016/s0010-7824(01)00248-7
169. Homeida MM, Eltoum IA, Ali MM, Suliaman SM, Elobied EA, Mansour M, et al. The effectiveness of annual versus biennial mass chemotherapy in reducing morbidity due to schistosomiasis: a prospective study in Gezira-Managil, Sudan. Am J Trop Med Hyg. 1996;54: 140–5. doi:10.4269/ajtmh.1996.54.140
170. Kamel MA, Miller FD, el Masry AG, Zakaria S, Khattab M, Essmat G, et al. The epidemiology of Schistosoma mansoni, hepatitis B and hepatitis C infection in Egypt. Ann Trop Med Parasitol. 1994;88: 501–9. doi:10.1080/00034983.1994.11812897
171. El-Khoby T, Hussein MH, Galal N, Miller FD. Epidemiology 1, 2, 3: origins, objectives, organization, and implementation. Am J Trop Med Hyg. 2000;62: 2–7. doi:10.4269/ajtmh.2000.62.2
172. Carlton EJ, Hsiang M, Zhang Y, Johnson S, Hubbard A, Spear RC. The impact of Schistosoma japonicum infection and treatment on ultrasound-detectable morbidity: a five-year cohort study in Southwest China. PLoS Negl Trop Dis. 2010;4: e685. doi:10.1371/journal.pntd.0000685
173. Liu J, Zhao G, Wu Z, Tao B, Jiang Q. The morbidity investigation of residents in a highly endemic village of schistosomiasis in Poyang Lake region. Zhongguo Ji Sheng Chong Xue Yu Ji Sheng Chong Bing Za Zhi. 1998;16: 197–200.
174. Nooman ZM, Hassan AH, Mishrirky AM, Ragheb M, Abu-Saif AN, Abaza SM, et al. The use and limitations of ultrasonography in the diagnosis of liver morbidity attributable to Schistosoma mansoni infection in community-based surveys. Mem Inst Oswaldo Cruz. 1995;90: 147–54. doi:10.1590/s0074-02761995000200004
175. Abdel Wahab MF, Esmat G. The value of ultrasonography in assessment of portal hypertension in hepatosplenic schistosomiasis. Mem Inst Oswaldo Cruz. 1992;87 Suppl 4: 143–7. doi:10.1590/s0074-02761992000800021
176. Souza M, De Toledo C, Borges D. Thrombocytemia as a predictor of portal hypertension in schistosomiasis. Dig Dis Sci. 2000;45: 1964–1970.
177. Otoni A, Antunes C, Tavares F, Araujo D, Pereira T, de Queiroz L, et al. Thrombocytopenia as a marker of liver steatosis in a low-endemic area for schistosomiasis mansoni. Rev Assoc Med Bras. 2017;63: 532–537. doi:10.1590/1806-9282.63.06.532
178. Drummond SC, Pereira PN, Otoni A, Chaves BA, Antunes CM, Lambertucci JR. Thrombocytopenia as a surrogate marker of hepatosplenic schistosomiasis in endemic areas for Schistosomiasis mansoni. Rev Soc Bras Med Trop. 2014;47: 218–22. doi:10.1590/0037-8682-0020-2014
179. Veiga ZST, Villela-Nogueira CA, Fernandes FF, Cavalcanti MG, Figueiredo FA, Pereira JL, et al. Transient elastography evaluation of hepatic and spleen stiffness in patients with hepatosplenic schistosomiasis. Eur J Gastroenterol Hepatol. 2017;29: 730–735. doi:10.1097/MEG.0000000000000853
180. Ohmae H, Tanaka M, Hayashi M, Matsuzaki Y, Kurosaki Y, Blas BL, et al. Ultrasonographic and serologic abnormalities in Schistosoma japonicum infection in Leyte, the Philippines. Am J Trop Med Hyg. 1992;46: 89–98. doi:10.4269/ajtmh.1992.46.89
181. Friis H, Ndhlovu P, Kaondera K, Franke D, Vennervald BJ, Christensen NO, et al. Ultrasonographic assessment of Schistosoma mansoni and S haematobium morbidity in Zimbabwean schoolchildren. Am J Trop Med Hyg. 1996;55: 290–294. doi:10.4269/ajtmh.1996.55.290
182. Abdel-Wahab MF, Esmat G, Farrag A, el-Boraey Y, Strickland GT. Ultrasonographic prediction of esophageal varices in Schistosomiasis mansoni. Am J Gastroenterol. 1993;88: 560–3.
183. Boisier P, Serieye J, Ravaoalimalala V, Roux J, Esterre P. Ultrasonographical assessment of morbidity in schistosomiasis-mansoni in madagascar - a community-based study in a rural-population. Trans R Soc Trop Med Hyg. 1995;89: 208–212. doi:10.1016/0035-9203(95)90498-0
184. Doehring-Schwerdtfeger E., Abdel-Rahim I.M., Mohamed-Ali Q., Elsheikh M., Schlake J., Kardorff R., et al. Ultrasonographical investigation of peripheral fibrosis in children with Schistosoma mansoni infection: Evaluation of morbidity. Am J Trop Med Hyg. 1990;42: 581–586. doi:10.4269/ajtmh.1990.42.581
185. Gerapacher-Lara R, Pinto-Silva RA, Rayes AA, Drummond SC, Lambertucci JR. Ultrasonography of periportal fibrosis in schistosomiasis mansoni in Brazil. Trans R Soc Trop Med Hyg. 1997;91: 307–9. doi:10.1016/s0035-9203(97)90087-0
186. Pereira LM, Domingues AL, Spinelli V, McFarlane IG. Ultrasonography of the liver and spleen in Brazilian patients with hepatosplenic schistosomiasis and cirrhosis. Trans R Soc Trop Med Hyg. 1998;92: 639–42. doi:10.1016/s0035-9203(98)90794-5
187. Cota GF, Pinto-Silva RA, Antunes CM, Lambertucci JR. Ultrasound and clinical investigation of hepatosplenic schistosomiasis: evaluation of splenomegaly and liver fibrosis four years after mass chemotherapy with oxamniquine. Am J Trop Med Hyg. 2006;74: 103–7.
188. Silva LC, Andrade LM, de Paula IB, de Queiroz LC, Antunes CM, Lambertucci JR. Ultrasound and magnetic resonance imaging findings in Schistosomiasis mansoni: expanded gallbladder fossa and fatty hilum signs. Rev Soc Bras Med Trop. 2012;45: 500–4. doi:10.1590/s0037-86822012005000008
189. Marinho CC, Nicolato AJPG, Reis VW, Dos Santos RC, Silva JC, Faria HP, et al. Ultrasound evaluation of schistosomiasis-related morbidity among the Xakriabá people in the state of Minas Gerais, Brazil. Radiol Bras. 2020;53: 7–13. doi:10.1590/0100-3984.2019.0047
190. Strahan R, Chiyesu KO, Schneider-Kolsky ME. Ultrasound study of liver disease caused by Schistosoma mansoni in rural Zambian schoolchildren. J Med Imaging Radiat Oncol. 2012;56: 390–7. doi:10.1111/j.1754-9485.2012.02371.x
191. Medhat A., Nafeh M., Swifee Y., Helmy A., Zaki S., Shehata M., et al. Ultrasound-detected hepatic periportal thickening in patients with prolonged pyrexia. Am J Trop Med Hyg. 1998;59: 45–48. doi:10.4269/ajtmh.1998.59.45
192. Houston S, Munjoma M, Kanyimo K, Davidson RN, Flowerdew G. Use of ultrasound in a study of schistosomal periportal fibrosis in rural Zimbabwe. Acta Trop. 1993;53: 51–8. doi:10.1016/0001-706x(93)90005-v
