# Supplementary material for: The role of point-of-care ultrasound in the assessment of schistosomiasis-induced liver fibrosis: A systematic scoping review
Source: PLoS Negl Trop Dis. 2024 Mar 20;18(3):e0012033. doi: 10.1371/journal.pntd.0012033 (PMC10954168; doi:10.1371/journal.pntd.0012033)
Supplement: S3 Text — The English translation of ultrasound protocol produced by the China CDC is provided. This is from pages 97–98 of the Chinese Schistosomiasis Control Manual. (DOCX) [file pntd.0012033.s005.docx]

China CDC guidance (2000):

0 = normal

1 = focal echodense areas, scattered within the liver parenchyma with absence of definite borders, specifically: evenly distributed echoes but stronger and coarser

2 = stronger light bands forming a fish-scale pattern, scattered focal echodense areas > 20mm in diameter, specifically: echo lacks evenness, coarse speck of light, scattered thin network-like echo visible within all parts of the liver, liver blood vessel wall echo strength and thicken slightly, hepatic vascularisation rather normal

3 = echodense bands forming a contiguous network, multiple focal echodense areas > 20 mm in diameter, masses with central fibrosis, specifically: uneven echo, coarsen speck of light, echo quite strong, large coarse network-like echo visible within all parts of the liver, portal vein wall thickens prominently, blood vessel lumen within liver becomes thin and narrow and unclear in display, liver size shrinks
