# Supplementary material for: The role of point-of-care ultrasound in the assessment of schistosomiasis-induced liver fibrosis: A systematic scoping review
Source: PLoS Negl Trop Dis. 2024 Mar 20;18(3):e0012033. doi: 10.1371/journal.pntd.0012033 (PMC10954168; doi:10.1371/journal.pntd.0012033)
Supplement: Attachment — Submitted filename: response_to_reviewers.docx [file pntd.0012033.s006.docx]

**RESPONSE TO REVIEWER COMMENTS**

**REVIEWER 1**

**Reviewer 1, comment 1:** Authors should include a Chinese or Chinese-speaking author to include the 29 excluded manuscripts in Chinese, which will more than double the articles pertaining to ultrasound (US) use in S. japonicum, and to appropriately describe the China CDC US guidelines.

***Response:*** Thank you for your comment. We acknowledge that a large number of studies have been excluded due to them being published in Mandarin. We appreciate that including them could radically change the statistics on the use of protocols for *S. japonicum,* which could currently under-represent the use of the China CDC guidance. Therefore, we have decided to reach out to a Mandarin-speaking student from China within our institute to screen and extract these studies as appropriate, and to include the relevant studies.

We looked again at the availability of the full-text of these studies, and 22 were available. After full-text screening, 16 were eligible for extraction. Therefore, we now have 41 studies that concern *S. japonicum.* This has doubled the number of studies in the review that use the guidance from the China CDC. Statistics and plots throughout the manuscript have been updated to include these studies. The methods have also been updated. The inclusion of these studies has allowed us to compare *S. japonicum* and *S. mansoni,* in particular with regard to the aims of the studies and the study designs (Results: Lines 280-281, 288-289). It has also allowed us to understand more comprehensively the guidance that has been produced by the China CDC. We now understand that there was a handbook published in 2000 (Department of Disease Control, Ministry of Health, China. Diagnosis of schistosomiasis. Handbook of schistosomiasis control. 3rd edition. Shanghai: Shanghai Press on Science and Technology; 2000:97-113.) which built upon the 1992 Cairo guidance, using a similar staging system and the same views. There was further guidance released in 2006, but this isn’t explicitly cited by studies in the review.

A supplementary file of the English translation of the staging system in the China CDC handbook (2000) is now provided to the benefit of other readers. This handbook is not available online in any format or language; the staging system was obtained through the translation (by HC) of a physical copy in China. Please refer to S3 Text for the translated staging system.

**Reviewer 1, comment 2:** Describe the excluded 34 manuscripts without full text and how it affects the review.

***Response:*** Thank you for your comment, it is important to consider the manuscripts that could not be found and their characteristics. We have added some information about the study country of the excluded manuscripts without full text to Figure 1. The number of excluded studies is now 42, having looked again at the Chinese language studies (see comment 1). Two of the 34 full texts that could not originally be found have now been obtained from the *Chinese Journal of Parasitology and Parasitic Diseases*. However, 10 of the Chinese language studies could not be found in full text, and had to be excluded at this stage.

Regarding the country of study, 22/42 studies that could not be found stated their study country explicitly in the abstract. 12/22 were conducted in China, 9/22 were conducted in Egypt, and 1/22 were conducted in Uganda. We also looked at the journal of the publications, and found that 15/20 studies that did not state the study country were published in an Egyptian journal. We also found that 21/42 studies were from before 2000. These distributions are the same as the included studies in the review. Egypt and China were the most common study countries in included studies, and therefore are already well represented in the review. Moreover, the studies that could not be found only concerned *S. mansoni* or *S. japonicum*, both of which are now well represented in the review. We conclude that the exclusion of these studies, to our knowledge, has little impact on the findings of the review.

**Reviewer 1, comment 3:** Overall, there is a limited analytical description of the studies, focusing only on their general features. Some sections of the Discussion can be expounded in the Results (Clinical translation, intra/inter-observer agreement). A brief mention in the Results on the performance of ultrasound as a diagnostic test for PPF, a synthesis on how the Cairo protocol evolved into the Niamey protocol, and elaboration on why Niamey is not being used for S. japonicum are suggested.

***Response:*** Thank you for this comment, we recognise that there are further aspects of the use of ultrasound in schistosomal PPF that could be acknowledged in the manuscript. For clinical translation, the use cases of ultrasound in schistosomal PPF are described in the study aims sections in the Methods and Results (Lines 178-193, 280-281, Table 1). Also, there is a quantitative description of inter- and intra-observer variation in the Results (Lines 391-398), and an expansion on this in the Discussion (Lines 604-633).

To address your comment on performance of ultrasound as a diagnostic test, we must first define what is meant by ‘performance’. This is difficult to define and to consolidate into a single statistic due to the heterogeneity in the studies’ evaluation of ultrasound as a diagnostic test. The heterogeneity primarily lies in the comparison test (which itself might be unreliable) and the protocol used to stage fibrosis using ultrasound. Agreement between transient elastography or biopsy and ultrasound imaging could be useful to evaluate ultrasound imaging against an ‘objective’ measure of fibrosis. Comments on these agreements have been included in the Results (Lines 319-327). Additionally, a comment on heterogeneity between the studies and the impact of this on enabling evaluation of ultrasound imaging as a diagnostic test is included in the Discussion (Lines 446-448).

The evolution of the Cairo protocol into the Niamey protocol is difficult to establish from the literature in this review alone. Furthermore, the published protocols do not make explicit the process and discussions that led to the final content. There are common characteristics that are considered in the Cairo and Niamey protocols such as periportal thickening, size of left and right liver lobes, portal vein diameter, presence of collateral vessels and gallbladder wall thickening. The key differences are a mention in the Niamey protocol that measurements of organ size should be height-adjusted and there should be reference measurements from the same population group; and the addition of the image patterns.

Thank you for your comment on the use of the Niamey protocol for *S. japonicum.* This may be because the China CDC released specific guidance for *S. japonicum* in the same year that it was released (2000). Also, the recommendation in the Niamey protocol is to use the guidance for *S. mansoni*, and signposts to the Cairo protocol for information specific to *S. japonicum.* The Niamey protocol also states that “Another workshop will review the protocols for *S. japonicum* and other species prevalent in Asia.” Whether this workshop has ever taken place is unclear to our knowledge.

**Reviewer 1, comment 4:** Table 1 shows that most studies focused on diagnostic agreement, but there were minimal details. Were staging systems being compared, or other procedures (Lines 296-9)? Elaboration on the performance of US on clinical severity and risk factors is also suggested.

***Response:*** Thank you for your comment. Diagnostic agreement encompasses both agreement between ultrasound staging systems and agreement between an ultrasound staging system and another non-ultrasound diagnostic test. We have now included more details on the agreement between ultrasound and biopsy, and transient elastography (see comment 3).

For the performance of ultrasound imaging as a predictor of a clinical outcome, we have 29 studies looked at a clinical outcome alongside performing ultrasound staging for periportal fibrosis. The different clinical outcomes in these studies were grades or bleeding of varices and portal hypertension (listed in Lines 281-282). In many cases it was difficult to establish what the link was between the clinical outcome and ultrasound staging was, due to ultrasound staging being used to calculate some clinical severity score combined with other variables, or poor reporting. This is a limitation of a scoping review: there is no risk of bias assessment so the collation of relationships between an exposure and an outcome is more challenging to perform reliably. Where the reporting was clear (only 8 studies), 6/7 studies found a positive association between fibrosis grade and variceal grade and 3/4 studies found a positive association between fibrosis grade and variceal bleeding.

**Reviewer 1, comment 5:** Please clarify Fig 4 and Line 305 for consistency on number of studies utilizing the Niamey protocol.

***Response****:* Thank you for this comment. In Line 305 (now Lines 329-330), we consider all studies when reporting the number that use the Niamey protocol. In Figures 4(a) and (b) we only consider studies that reported on study year and studies that were on *S. mansoni* and *S. japonicum* respectively. This is clarified in the caption of Figure 4. For clarification and interest, we include here a graph showing the use of the different staging systems across all 192 studies.

**Reviewer 1, comment 6:** Was data available to assess whether certain ultrasound machines perform better than others?

***Response:*** Thank you for this comment. None of the studies used more than one machine to allow a within study comparison, and due to the heterogeneity in the study aims and protocols used it was not possible to do a between study comparison to assess the performance of the different machines. We hypothesise that bigger differences in performance will likely be due to the use of either static or portable machines, used in facility or community settings respectively. Static machines are able to acquire higher quality images, however these cannot be taken into community-based settings for practical reasons. In terms of guidance, a recommendation on a machine to use should be based on the use case and the resources available. We have added a comment on ultrasound machines in the Discussion (Lines 596-603) to address this.

**Reviewer 1, comment 7:** The conclusions are sound, however the statement that there is "difficulty in choosing a reference standard for schistosomal PPF staging" (378) may be unsupported given that 70% of the studies on S. mansoni after 2000 have adopted the WHO-recommended Niamey protocol.

***Response:*** Thank you for your comment. It is true that within ultrasound staging of schistosomal PPF after 2000, there is a wide consensus that the Niamey protocol is used. This consensus, however, is not equivalent to a reference standard. A reference standard must be clinically validated and be reproducible among different populations. More studies need to be done applying the same methods in different populations to reproduce similar outcomes to ensure that the Niamey protocol patterns are sufficient to be a reference standard. To address this, some clarifications have been made in the Discussion (Lines 411-416), which better define what is required for a reference standard.

**Reviewer 1, comment 8 and 9:** The manuscript is easy to follow and understand.

The manuscript is an expansive review of the literature on ultrasound for hepatosplenic schistosomiasis. The manuscripts describes the different aims, populations, study settings and species of studies on ultrasound for schistosomiasis. A historical perspective of the various staging platforms is noteworthy.

***Response:*** We thank the reviewer for acknowledging the strengths of the manuscript and for their thoughtful comments.

**REVIEWER 2**

**Reviewer 2, comment 1:** Study is well described and the value of performing a scoping review is well articulated. The study design appropriately addresses the stated objective and question stated. The article selection criteria, review and selection are well described. No ethical considerations in this review.

***Response:*** We thank the reviewer for acknowledging the strengths of the manuscript and for their thoughtful comments.

**Reviewer 2, comment 2:** Results are clearly presented. Given the quantity of data that the authors abstracted there is only a limited amount of this data presented. It would be valuable to add some additional data analysis. I would suggest looking at who is obtaining the ultrasound images. I realize not all of the studies report on the sonographer but this would be valuable information to add for the studies that do report this.

***Response****:* Thank you for your comment. We have added a list of occupations of the ultrasound practitioner that were reported (Lines 383-384). We have also attempted to collect additional information on who is obtaining the ultrasound images by recording the affiliations of the sonographer, whether they are local to the endemic area, and whether the study had any capacity building element to train local sonographers. The studies that were looked at again were the 72 studies that reported on the occupation of the acquirer of the ultrasound images. For the most part (43/72), studies did not report any additional information on the person/people collecting ultrasound images, other than their occupation. When the acquirer of the ultrasound images had an affiliation (23/72 studies), this was with a university in the study country in 17/23 studies. However, local capacity building for sonographers was only explicitly mentioned in one study. Additionally, there is likely to be reporting bias in these statistics since people without university affiliations are missed out.

In terms of the relevance of this to the development of the Niamey protocol, who is using the guidance should shape its development. It is clear that both researchers and clinicians are using the guidance and therefore it should be adaptable to both contexts. For a clinical context, this could mean refining and simplifying the guidance so that it is feasible to carry out the whole protocol quickly and accurately and perhaps even suitable for someone who is not a highly trained sonographer (given the limited technical expertise in low resource settings).

**Reviewer 2, comment 3:** Conclusion is well supported by the data presented. The authors are clear in discussing what the data suggests about the current state of ultrasound for intestinal schisto and link this to next steps in how to advance our understanding in this area. Public health value is clearly addressed.

***Response:*** We thank the reviewer for acknowledging the conclusions and public health value of our manuscript.

**Reviewer 2, comment 4:** In this study authors perform a scoping review of the current literature on the use of ultrasound for intestinal schistosomiasis and heterogeneity in what type of protocol (if any) studies adhere to. This is a well done scoping review that presents important data and highlights a known but significant gap in the application of ultrasound for this disease pathology. As noted above the quantity of data the authors abstracted could allow or additional data around image acquisition specifics to be reported in the results section. The authors should expand their limitations section especially around Mekongi and Japonicum species - given the number of mandarin studies that were excluded this scoping review primarily tells us about Mansoni studies. This does not diminish the author’s conclusions but it is worth highlighting especially if new consensus about ultrasound protocols are to be considered.

***Response:*** Thank you for your comment and for acknowledging the importance of the data presented. With regard to *S. japonicum,* we have now included a Mandarin-speaking co-author who screened and extracted the studies in Mandarin as appropriate.

We looked again at the availability of the full-text of these studies, and 22 were available. After full-text screening, 16 were eligible for extraction. Therefore, we now have 41 studies that concern *S. japonicum.* This has doubled the number of studies in the review that use the guidance from the China CDC. Statistics and plots throughout the manuscript have been updated to include these studies. The inclusion of these studies has allowed us to compare *S. japonicum* and *S. mansoni,* in particular with regard to the protocol usage and the aims of the studies. This has allowed us to conclude that these species should certainly be considered separately when producing new guidance. It has also allowed us to understand more comprehensively the guidance that has been produced by the China CDC. We now understand that there was a handbook published in 2000 (Department of Disease Control, Ministry of Health, China. Diagnosis of schistosomiasis. Handbook of schistosomiasis control. 3rd edition. Shanghai: Shanghai Press on Science and Technology; 2000:97-113.) which built upon the 1992 Cairo guidance, using a similar staging system and the same views. There was further guidance released in 2006, but this isn’t explicitly cited by studies in the review.

Regarding *S. mekongi,* we appreciate that this is a tiny minority of studies in the review. There were no studies excluded that considered *S. mekongi*, therefore it can be concluded that there is extremely limited literature on this species. This may be due to a very small distribution, a lower prevalence or less research being done in the affected countries (Cambodia, Laos and Thailand). We have noted this in the Limitations section (Lines 751-754).

**Reviewer 2, comment 5:** The biggest gap in this study that needs to be addressed is the area of machine learning. The authors do not even consider this application and their study lacks future relevance by excluding this topic. There is a growing use of machine learning and “auto grading” of imaging studies by computers. More than the need to standardize for human reviewers there is an urgent need to create a consensus around schistosomiasis scoring systems before computers are programmed to read and interpret these images based on an outdated protocol. The authors should expand their conclusion to address this issue moreover concisely and directly. A brief review to see if any studies have already been published around machine learning or AI and schisto would add strength to the study conclusions and the call for an updated consensus protocol.

***Response*:** Thank you for this comment, we agree that machine learning will be applied in the future of this field and that standardisation is deeply important for the reliability of automated grading tools. Although none of the studies in the review use or address machine learning as being a future direction for schistosomal PPF grading, it has been used in other areas of schistosomiasis research and there is current work ongoing to create a tool for schistosomal PPF staging. The areas of machine learning covered in limited scope for schistosomiasis are egg counting, reading of POC-CCA rapid diagnostic tests, and the classification of lesions caused by female genital schistosomiasis (FGS). However, there is currently no published research on the staging of schistosomal PPF using machine learning. Machine learning for ‘auto-grading’ is ongoing in the SchistoTrack Cohort led by the authors here (EO, JAN, GFC). To share protocol considerations needed to enable the future of machine learning models and to acknowledge the current work on machine learning in other areas of schistosomiasis and other liver disease, we have added an amendment to the Discussion (Lines 642-654).
